# Closing the Translational Gap in Tuberculosis Lipidomics for Resource-Constrained Settings

**DOI:** 10.3390/metabo16100708

**Published:** 2026-09-24

**Authors:** Joash Gombe, Gabriel Dida, Noel Onyango

**Affiliations:** 1 Public Health Institute, Faculty of Health, Liverpool John Moores University, Liverpool L3 3AF, UK; 2 Department of Medical Microbiology and Immunology, Faculty of Health Sciences, University of Nairobi, Nairobi 00100, Kenya

**Keywords:** tuberculosis, lipidomics, biomarkers, diagnostics, PRISMA-ScR, cohorts, validation, LMICs, cough aerosol

## Abstract

Tuberculosis (TB) host lipidomics research has produced a technically sophisticated evidence base that remains structurally unprepared for clinical translation. Discovery-phase, cross-sectional studies dominate the field; longitudinal and independently validated studies remain scarce; and no study has yet linked a host plasma lipid signature to a direct aerobiological measure of infectiousness, such as Cough Aerosol Culture (CAC) positivity. We argue that closing this gap requires a deliberate reorientation of the field: from single-time-point diagnostic discovery toward prospective, longitudinal cohort designs; from reliance on systemic blood-based matrices alone toward non-invasive, point-of-care-compatible specimens such as urine and exhaled breath; and from fragmented, idiosyncratic reporting toward standardized frameworks that allow findings to be pooled and compared across studies. We further argue that low- and middle-income countries (LMICs), which host the majority of the existing TB lipidomics evidence base and carry the greatest burden of disease, are well positioned to lead this next phase rather than wait for it to be imported. This Perspective sets out the case for that reorientation and proposes a concrete translational agenda built around three pillars: longitudinal validation, a point-of-care specimen shift, and reporting standardization.

## 1. The Technical Maturity Trap in TB Lipidomics

There is a productive tension at the heart of tuberculosis (TB) host lipidomics research. To systematically evaluate the state of this field, we recently conducted a Preferred Reporting Items for Systematic Reviews and Meta-Analyses Extension for Scoping Reviews (PRISMA-ScR) [1] registered companion scoping reviews (https://doi.org/10.17605/OSF.IO/5EGMR) (File S2) [2,3,4,5,6,7,8,9,10,11,12,13,14,15] mapping 48 primary empirical lipidomics studies published between 2014 and 2026 (Table S1) [16,17,18,19,20,21,22,23,24,25,26,27,28,29,30,31,32,33,34,35,36,37,38,39,40,41,42,43,44,45,46,47,48,49,50,51,52,53]. The resulting dataset reveals a clear paradox: while the field has achieved substantial technical sophistication, it remains caught in a structural standstill that blocks clinical translation (Table S2, Figure S1).

On one hand, high-resolution liquid chromatography–tandem mass spectrometry (LC-MS/MS) platforms are now regularly deployed across global research hubs to profile hundreds of distinct lipid species from nanogram quantities of plasma. Reproducible molecular signatures are emerging with moderate consistency across geographically diverse cohorts, particularly involving systemic alterations in glycerophospholipids and sphingolipids, including phosphatidylcholines, lysophosphatidylcholines, ceramides, and sphingomyelins [54,55,56,57].

On the other hand, this technical maturity coexists with a marked absence of the prospective, longitudinal, and validation-stage evidence required for clinical deployment. Cross-sectional discovery studies dominate the field, prospective studies linked to treatment outcomes are consistently described as lacking, and longitudinal validation with long-term follow-up remains necessary before lipidomic biomarkers can be used in practice [58,59,60].

We term this paradox the “technical maturity trap”: a state in which a field has refined its discovery machinery to a high degree but has systematically failed to invest in the infrastructure needed to deploy its outputs. From our recent review, the quantitative parameters of this trap are clear (File S1): LC-MS/MS is utilised at 77.1% frequency across the literature, while 64.6% (*n* = 31) of studies rely exclusively on systemic blood-based matrices ill-suited for point-of-care environments, 70.8% completely lack independent cohort validation, and 64.6% (*n* = 31) remain confined to single-time-point diagnostic discovery. Only 12.5% of the global evidence base addresses treatment monitoring or prognosis [60].

“The TB lipidomics field has built an extremely sophisticated discovery engine. The urgent task now is to build the translational road that leads from molecular signal to clinical tool.”

This Perspective is not a critique of discovery science (the diagnostic studies that dominate the current evidence base have been necessary and productive); rather, it is a call for a deliberate, coordinated reorientation toward the next phase of the translational pipeline, one that will require different study designs, different specimen types, and geographically diverse institutional partnerships than those that characterised the discovery phase.

Figure 1 summarizes the conceptual model motivating this agenda: a plasma host lipid signature that tracks disease activity across the infection spectrum and, at its most active end, the divergence between infectious and non-infectious disease.

## 2. Three Structural Deficits Blocking Translation

### 2.1. The Longitudinal Deficit

Tuberculosis is a disease with a minimum treatment duration of six months. The full clinical trajectory of a patient, from pre-treatment presentation through bacterial clearance, immune reconstitution, and the risk of late relapse, spans years. Yet the TB lipidomics evidence base, which is 47.9% case–control and largely cross-sectional, has no memory of this trajectory. It captures patients at a single diagnostic moment and describes their metabolic state at that point. It does not know what happened to them afterward.

This matters profoundly for several reasons. First, without longitudinal lipidome data collected serially across the treatment course, it is impossible to determine whether identified biomarkers, however robust they appear at diagnosis, can distinguish patients progressing toward cure from those trending toward treatment failure.

Second, the current case–control paradigm captures patients exclusively at the diagnostic moment, producing a static picture of a deeply dynamic biological process. Lipid mediators of inflammation and resolution, namely prostaglandins, lipoxins, and resolvins, are rapidly metabolised molecules whose dynamic signalling roles cannot be adequately captured in a single blood draw. Studies such as [61] Chen et al. [58] and Anh et al. [59], which followed patients across the treatment course and identified lipidome alterations correlating with treatment phase, demonstrate that longitudinal lipidomic profiling is analytically feasible. Neither study, however, was powered to predict treatment failure or relapse, the clinical endpoints that matter most for patient management, underscoring that feasibility has been demonstrated but the critical translational step remains undone.

Third, the six-month standard treatment regimen provides a clinically defined temporal scaffold for biomarker discovery that is structurally analogous to a pharmacokinetic study where there is a defined intervention, a defined duration, and defined clinical outcomes (cure, treatment failure, relapse). This scaffold is being systematically underutilized.

The 14 longitudinal studies (29.2%) in the current evidence base demonstrate that such work is feasible and publishable.

### 2.2. The Specimen Matrix Deficit

Tuberculosis is a pulmonary disease, yet 64.6% of TB lipidomics studies measure lipids in blood-based matrices, systemic proxies that reflect the whole-body metabolic response to infection rather than the local biochemistry at the site of pathology [60]. Blood is a distant echo of the lung. The lipids measured in plasma after passing through hepatic first-pass metabolism, dilution into the systemic circulation, and remodelling by circulating enzymes are several metabolic steps removed from the lipid environment of the infected macrophage, the granuloma, and the alveolar space from which aerosols are generated.

This anatomical distance is not, however, a fundamental barrier to clinical utility; systemic lipid remodelling is a well-established feature of active TB, and the plasma lipidome integrates host immune–metabolic signals from across the body, including from the pulmonary compartment [41,54,55]. The critical question is not whether plasma lipids carry disease-relevant information—the evidence base confirms that they do—but whether the specific disease dimensions that matter most clinically have been adequately investigated using this matrix.

Current evidence does not support that claim. Lipid biomarkers identified in plasma have been evaluated almost exclusively for their capacity to distinguish active TB from healthy controls at a single diagnostic time point [60]. Their capacity to distinguish TB from other respiratory conditions producing overlapping systemic lipid perturbations, pneumonia, lung cancer, and chronic obstructive pulmonary disease remains incompletely characterised [62,63,64]. More critically, no study in the current evidence base has linked plasma lipid profiles to direct aerobiologic measures of infectiousness, such as Cough Aerosol Culture (CAC) positivity, the most direct available phenotypic measure of an individual patient’s transmission potential [60]. This is not a statement about the biological implausibility of such a relationship. *Mycobacterium tuberculosis* (Mtb) infection produces systemic immune–metabolic remodelling that is detectable in plasma; it is entirely plausible that the intensity or character of this remodelling correlates with bacterial burden and aerosol generation capacity. It is simply a relationship that has never been investigated. Closing this specific gap, correlating plasma lipid signatures with CAC-defined infectiousness in prospective transmission cohorts, represents one of the most consequential and tractable frontiers in TB biomarker science.

The practical case for non-invasive specimen types remains important in parallel. For point-of-care deployment in community health worker settings, phlebotomy is a substantial barrier to scale. The 3 identified exhaled breath studies and 4 urine studies in the recent review represent early-stage evidence for alternative matrix feasibility [60], but, as discussed above, methodological heterogeneity across collection approaches currently prevents meaningful cross-study synthesis. Pre-analytical standardization, reference range development, and validation against culture-confirmed TB status in diverse cohorts remain essential prerequisites before non-invasive lipidomics can progress beyond proof-of-concept.

### 2.3. The Validation Deficit

The finding that 70.8% of the evidence base lacks independent cohort validation is perhaps the single most consequential statistic in the current literature [60]. Internal cross-validation, the common alternative, is well known to generate optimistic performance estimates that fail to replicate in external cohorts, particularly when sample sizes are modest, and feature spaces are high-dimensional, as is inherently the case in untargeted lipidomics [65]. To illustrate, a hypothetical biomarker panel returning an internally validated AUC (Area Under the Curve) of 0.92 should be regarded with appropriate scepticism until tested in an independent cohort with different demographic characteristics, different TB strain backgrounds, and different comorbidity profiles.

This validation deficit has a geographic dimension. The centralisation of the evidence base within 3 LMIC nations—China (52.1%), South Africa (approximately 18.8%), and India (approximately 14.6%)—means that even internally consistent findings from these hubs may not generalize to the TB burden settings of Eastern Europe, Francophone Africa, or Southeast Asia [60]. Regional variations in host genetics, nutritional status, Body Mass Index (BMI), and Mtb strain lineage are each known to influence the host lipidome [66,67,68], yet their effect on biomarker generalisability has not been systematically studied. Until multi-site validation studies deliberately include these under-represented high-burden settings, the clinical deployability of identified lipid signatures will remain uncertain.

A related confounder deserves explicit consideration. In LMIC settings such as India, where neonatal Bacillus Calmette–Guérin (BCG) vaccination coverage approaches universal, BCG exposure itself induces measurable metabolic and immune reprogramming. Whether this baseline reprogramming narrows or is mistaken for the separation between active TB cases and healthy controls in plasma lipidomic comparisons has not been examined in the existing evidence base. Future validation cohorts should record BCG vaccination status as a covariate and, where feasible, stratify analyses by it, rather than assume that control groups are metabolically neutral.

A further question concerns LTBI. Three-arm comparisons of active TB, LTBI, and uninfected controls have been reported, and several describe lipid species that separate the three states [69]. The limitation is not that the comparison has been neglected, but that it has rarely been validated. Reported LTBI-discriminating signatures derive largely from single cohorts, and the validation deficit described above applies to them with particular force: LTBI prevalence, BCG exposure, and comorbidity structure vary sharply between settings, and each plausibly shifts the baseline against which latency is defined. The more consequential gap is prognostic. Classifying prevalent LTBI is of limited clinical value compared with identifying the small subset who will progress to active disease, and that question requires longitudinal follow-up of LTBI cohorts rather than further cross-sectional three-arm contrasts.

## 3. A Translational Agenda: From Discovery to Deployment

Closing the translational gap in tuberculosis lipidomics requires a structured, multi-phase research agenda. We propose three prioritised recommendations, each addressing one of the structural deficits identified above.

### 3.1. The Longitudinal Imperative

The design template for the next generation of TB lipidomics studies should be the prospective treatment cohort. Patients should be enrolled at the time of TB diagnosis confirmation, with baseline lipidomic profiling of plasma, and where feasible, urine and exhaled breath. Serial sampling should occur at clinically meaningful time points: 2 weeks (the period of maximal early bactericidal activity [70]), 2 months (the end of the intensive treatment phase), 6 months (end of treatment), and 12 months (late relapse assessment). Primary endpoints should include two-month culture conversion, treatment outcome at six months (World Health Organization defined cure, treatment failure, default), and relapse within 12 months of treatment completion.

This design directly addresses the most urgent clinical question: can the lipidome tell us, at diagnosis or at the end of the intensive phase, which patients are on a trajectory toward cure and which are not? The answer to this question has the potential to transform TB clinical management by enabling adaptive treatment strategies, extending treatment for patients whose lipid profiles predict relapse risk, or validating treatment completion for patients whose profiles confirm metabolic resolution [55].

### 3.2. The Point-of-Care Specimen Shift

For lipidomics to contribute to TB control at the point of need, i.e., in community health centres, district hospitals, and high-transmission settings, the specimen collection pathway must be radically simplified. The technical pathway from exhaled breath condensate to a quantitative lipid readout using portable mass spectrometry or lateral flow immunoassay technology is scientifically plausible and has precedent in adjacent fields [71]. The LMIC-specific precedent identified in this review, namely that immunoassays and gas chromatography–mass spectrometry (GC-MS) platforms are already deployed exclusively within LMIC research settings [60], demonstrates that the technology adaptation capacity exists. What is required is deliberate co-design of simplified analytical workflows with front-line health workers and district health system managers, not only with academic researchers.

We propose that exhaled breath aerosol lipidomics deserves particular investment. The study by Chen et al. [72] demonstrated that non-volatile organic compounds in exhaled breath particles correspond to active TB with analytical feasibility in a high-burden LMIC setting. Crucially, exhaled breath is both the output medium of TB transmission (aerosol droplet nuclei) and a potential diagnostic matrix. A biomarker that could simultaneously indicate active pulmonary TB and correlate with aerosol generation would represent a breakthrough for both clinical management and public health intervention.

### 3.3. The Standardization Imperative

The fragmentation of the TB lipidomics literature is an active barrier to translation. When lipid species are reported using inconsistent nomenclature, when extraction protocols are not standardised or reported in sufficient detail for reproduction, and when cohort characteristics are described using incompatible variables, meta-analytic synthesis becomes impossible, and biomarker validation networks cannot function. The field must converge on minimum reporting standards standards equivalent to the Minimum Information About a Microarray Experiment (MIAME), or the Lipid Metabolites and Pathways Strategy (LIPID MAPS) lipid nomenclature framework) [73,74,75,76,77].

Practically, this requires that all TB lipidomics manuscripts report (1) the complete LIPID MAPS species identifier for all reported significant lipids; (2) the full pre-analytical and analytical protocol, including extraction solvent system, column chemistry, ion source parameters, and data processing pipeline; (3) the cohort’s comorbidity profile, including Human Immunodeficiency Virus (HIV) status and diabetes metrics; (4) specimen collection conditions, including fasting status and time from collection to processing; and (5) the statistical approach to multiple comparisons correction and cross-validation. Several journals are well positioned to enforce these standards through Supplementary Material requirements. These three priorities are summarized in Table 1.

## 4. The LMIC Opportunity: Leading the Next Phase

A striking feature of the TB lipidomics landscape is that the high-burden LMICs that bear the greatest need for improved diagnostics and monitoring tools are also the countries that have generated the majority of the existing evidence. China, India, and South Africa are not passive recipients of externally developed diagnostic technology but are active producers of the foundational science upon which such technology must be built. This is a strategic asset that is insufficiently recognized in global TB research funding narratives.

This implies that LMICs should not wait for high-income country (HIC) investigators to develop and then transfer longitudinal validation protocols. The infrastructure for prospective cohort studies exists in cities like Nairobi, Chennai, Cape Town, and São Paulo. The patient populations that would provide the most clinically relevant validation data—high HIV co-infection rates, high TB burden, multidrug-resistant tuberculosis strains (MDR-TB), and diverse nutritional profiles—are present in these settings, not in Boston or London. The institutional capacity at organizations such as the Kenya Medical Research Institute (KEMRI), the National Institute for Research in Tuberculosis (NIRT) in Chennai, and the South African Medical Research Council is real and growing.

What is currently missing is not capacity but coordination. A formal TB Lipidomics Validation Consortium, linking LMIC hubs across Sub-Saharan Africa, South Asia, Southeast Asia, and Latin America with harmonized protocols, shared reference standards, and jointly analysed datasets, would transform the current fragmented evidence base into a globally generalized and clinically deployable biomarker portfolio. The modeling for such consortia exists in the Global Tuberculosis Network and the Foundation for Innovative New Diagnostics (FIND) validation programs [78,79]; the scientific infrastructure for TB lipidomics is mature enough to warrant an equivalent initiative.

## 5. Conclusions

TB lipidomics stands at an inflection point. A technically mature diagnostic discovery phase has established that lipids, particularly glycerophospholipids and sphingolipids, carry disease-relevant information in plasma, breath, urine, and tissue of individuals with TB. The next phase requires that this information be tested over time, in diverse populations, against clinical endpoints that matter for TB control, not only in terms of diagnosis but also treatment response, patient outcomes, and transmission.

The three structural deficits identified here, namely the longitudinal deficit, the specimen matrix deficit, and the validation deficit, are not immutable features of the field. They are the product of funding priorities, methodological inertia, and the natural tendency of discovery science to optimise for novelty over replication. Addressing them requires deliberate choices: to fund prospective cohort studies rather than additional cross-sectional discovery efforts; to expand the range of clinical questions asked of the plasma lipidome rather than defaulting to diagnostic case–control designs; and to build multi-site validation networks that ensure findings generalize beyond the three LMIC hubs that currently dominate the evidence base.

Of all the gaps identified, one stands apart in both its novelty and its public health consequence. No study in the current evidence base has asked whether a patient’s plasma lipid profile correlates with their capacity to generate infectious aerosols. This is not a biologically implausible question; Mtb infection produces measurable systemic immune–metabolic remodelling detectable in plasma, and it is entirely plausible that the character or intensity of this remodelling reflects bacterial burden and aerosol generation capacity. It is simply a question that has never been investigated. Prospective studies correlating host plasma lipid signatures with Cough Aerosol Culture-defined infectiousness in transmission cohorts would directly address this gap and, if validated, would represent a transformative tool for identifying the most infectious index cases in high-burden urban settings where CAC itself lacks the scalability for routine deployment.

For institutions operating in high-burden LMIC settings, particularly those at the intersection of high TB prevalence, documented transmission hotspots, and established research infrastructure, such as Kenya Medical Research Institute’s Centre for Respiratory Diseases Research (KEMRI/CRDR) in Nairobi, this agenda represents not a burden but a generational scientific opportunity. The molecular foundation has been built. The clinical question is defined. The translation is now within reach, but only if the global TB research community makes the deliberate, coordinated investment required to complete it.

## Figures and Tables

**Figure 1:**
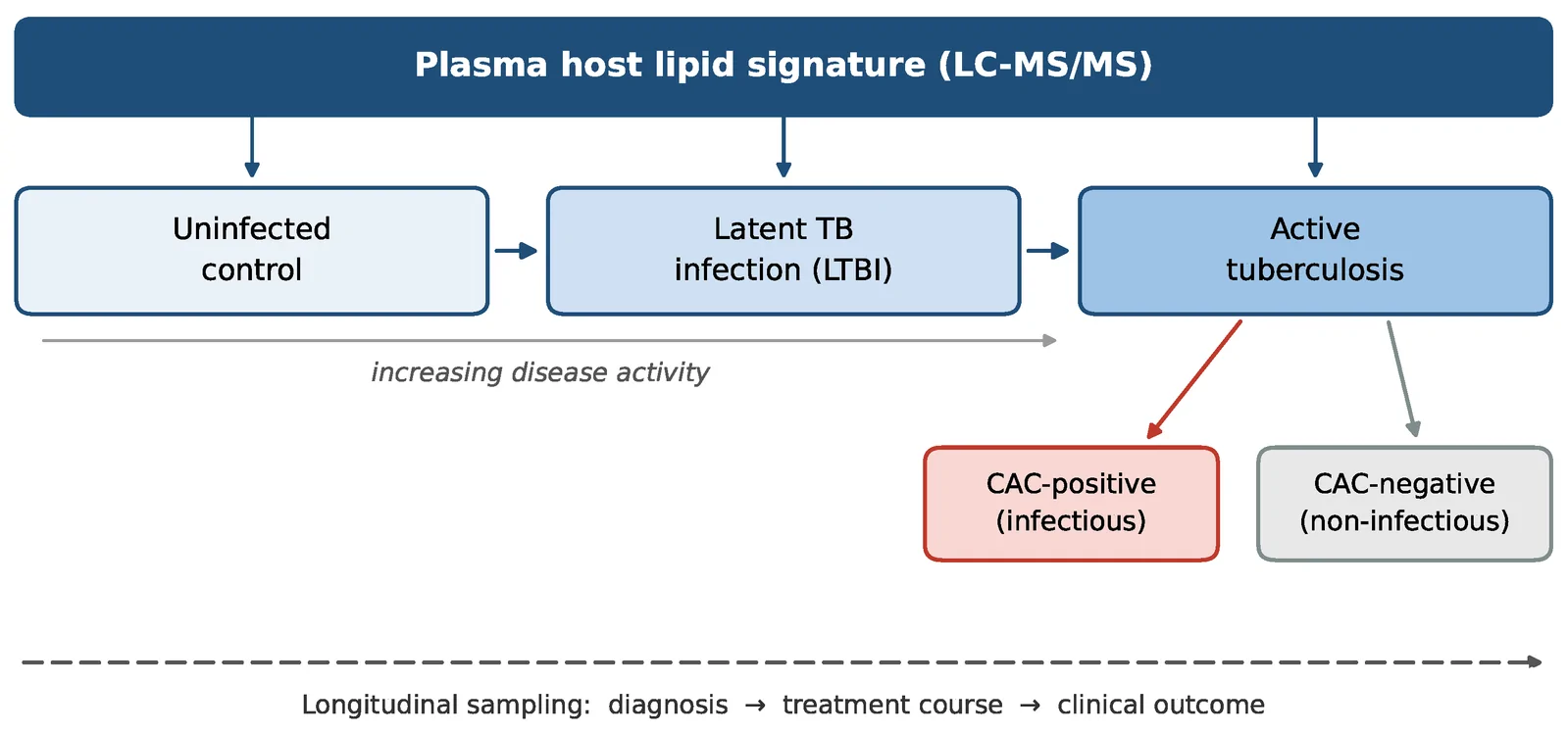
Conceptual model linking the plasma host lipid signature to disease activity (uninfected control → Latent Tuberculosis Infection (LTBI) → active tuberculosis) and, within active disease, to aerobiological infectiousness as defined by Cough Aerosol Culture (CAC) status. The dashed arrow indicates the longitudinal sampling dimension proposed in Section 3.1.

**Table 1:** Strategic pillars and actionable research priorities for closing the translational gap in tuberculosis lipidomics.

Strategic Pillar	Actionable Research Priorities
The Longitudinal Imperative	Launch multi-site prospective cohort studies with serial lipidome profiling across the full 6-month TB treatment course (endpoints: culture conversion, treatment failure, 6-month relapse). Link host lipid profiles to aerobiological measures of infectiousness via Cough Aerosol Culture (CAC) in dedicated transmission studies. Prioritize comorbidity-stratified analyses, focusing heavily on TB-HIV and Tuberculosis-Type 2 Diabetes Mellitus (TB-T2DM) phenotypes in all multi-site designs
The Point-of-Care Specimen Shift	Develop and validate non-invasive specimen collection and analysis protocols for exhaled breath and urine lipidomics with standardized pre-analytical workflows. Develop paediatric-specific lipidomic reference ranges and biomarker panels to address the gap in children under 15.
The Standardization Imperative	Adopt LIPID MAPS species-level nomenclature for all reported lipids. Report the complete pre-analytical and analytical protocol: extraction solvent system, column chemistry, ion source parameters, and data processing pipeline. Report cohort comorbidity profile, including HIV and diabetes status. Report specimen collection conditions, including fasting status and time from collection to processing. Report the statistical approach to multiple-comparison correction and cross-validation.

## Data Availability

No new data were created or analysed in this study. The scoping review protocol underpinning this Perspective is publicly registered at the OSF: https://doi.org/10.17605/OSF.IO/5EGMR.

## Supplementary Materials

The following supporting information can be downloaded at https://www.mdpi.com/article/10.3390/metabo16100708/s1, Table S1: Full list of included studies (*n* = 48); Table S2: Complete database search log; Figure S1: PRISMA-ScR flow diagram; File S1: Data extraction form; File S2: Open Science Framework (OSF)-registered protocol.

## Abbreviations

The following abbreviations are used in this manuscript:

Abbreviation	Full Term
TB	Tuberculosis
LC-MS/MS	Liquid Chromatography–Tandem Mass Spectrometry
GC-MS	Gas Chromatography–Mass Spectrometry
LMICs	Low- and Middle-Income Countries
HIC	High-Income Country
HIV	Human Immunodeficiency Virus
BMI	Body Mass Index
BCG	Bacille Calmette-Guérin
CAC	Cough Aerosol Culture
PRISMA-ScR	Preferred Reporting Items for Systematic Reviews and Meta-Analyses Extension for Scoping Reviews
OSF	Open Science Framework
AUC	Area Under the Curve
KEMRI/CRDR	Kenya Medical Research Institute, Centre for Respiratory Diseases Research
LIPID MAPS	Lipid Metabolites and Pathways Strategy
FIND	Foundation for Innovative New Diagnostics
WHO	World Health Organization
MDR-TB	Multidrug-Resistant Tuberculosis
LTBI	Latent Tuberculosis Infection
TB-HIV	Tuberculosis-HIV Coinfection
TB-T2DM	Tuberculosis-Type 2 Diabetes Mellitus Comorbidity
NIRT	National Institute for Research in Tuberculosis
MIAME	Minimum Information About a Microarray Experiment
