# Supplementary material for: Closing the Translational Gap in Tuberculosis Lipidomics for Resource-Constrained Settings
Source: Metabolites. 2026 Sep 24;16(10):708. doi: 10.3390/metabo16100708 (PMC13706928; doi:10.3390/metabo16100708)
Supplement: Supplementary file 1 [file metabolites-16-00708-s001.zip › Figure S1 PRISMA-ScR flow diagram.pdf]

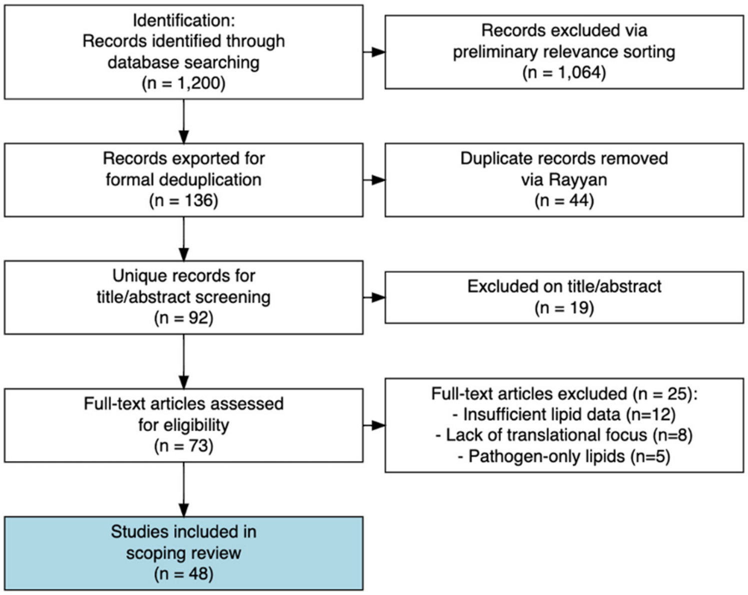

Figure S1 *The PRISMA-SCR flow diagram of the study selection process. The diagram illustrates the systemic flow of records from initial identification (n=1,200) to the final inclusion of 48 studies. Preliminary relevance sorting and duplicate resolution were performed to refine the search results prior to formal title/abstract and full text screening based on pre-defined eligibility criteria.*

The PRISMA-SCR flow diagram of the study selection process. The diagram illustrates the systemic flow of records from initial identification (n=1,200) to the final inclusion of 48 studies. Preliminary relevance sorting and duplicate resolution were performed to refine the search results prior to formal title/abstract and full text screening based on pre-defined eligibility criteria.
