# Supplementary material for: Closing the Translational Gap in Tuberculosis Lipidomics for Resource-Constrained Settings
Source: Metabolites. 2026 Sep 24;16(10):708. doi: 10.3390/metabo16100708 (PMC13706928; doi:10.3390/metabo16100708)
Supplement: Supplementary file 1 [file metabolites-16-00708-s001.zip › File S1 Data extraction form.pdf]

Supplementary Data Sheet S1. Data extraction form

Section	Variable	Description	Data Entry
**Section 1: General Study Information**	Paper ID		
	Title		
	Journal		
	Geographic Origin		
**Section 2: Study Characteristics**	Study Design		
	Primary Focus		
	Population Group		
	TB Disease State		
	Site of Disease (Pulmonary / Extrapulmonary / Both / Not specified / Not applicable)		
	Comorbidities		
**Section 3: Methodology & Analytical Platform**	Specimen Type		
	Sample Size (n)		
	Analytical Platform		
	Lipid Classes and candidate biomarkers identified		
**Section 4: Outcomes & Critical Synthesis**	Key Findings		
	Identified Gap		
	Reviewer Notes		
