# Supplementary material for: Closing the Translational Gap in Tuberculosis Lipidomics for Resource-Constrained Settings
Source: Metabolites. 2026 Sep 24;16(10):708. doi: 10.3390/metabo16100708 (PMC13706928; doi:10.3390/metabo16100708)
Supplement: Supplementary file 1 [file metabolites-16-00708-s001.zip › metabolites-4488496-File S2 Open Science Framework (OSF)-registered protocol.pdf]

Lipidomics and Tuberculosis Biomarker Discovery: A Scoping Review Proposal of Human, Animal, and In Vitro Evidence

Joash Odhalo Gombe

R2403D17764482

Research Methods LJMU-7505-PUBUNI-91123

28th September, 2025

Word Count - 2304

Introduction

Background

Tuberculosis (TB) remains one of the leading infectious causes of death worldwide, with an estimated 10.6 million new cases and 1.3 million deaths in 2022 (WHO, 2024). These burdens fall disproportionately on vulnerable populations, including children, people living with HIV, and communities in resource-limited settings, where diagnostic gaps remain a persistent barrier to timely care resulting in persistently high prevalence rates especially in high burden settings(Ong'ang'o *et al.*, 2025). Recent TB research increasingly uses large-scale biological profiling methods (omics) to generate new insights into disease processes and potential biomarkers. These include genomics (the study of genes), transcriptomics (gene activity), proteomics (proteins), and metabolomics (small metabolically active molecules). Within this family, lipidomics, the comprehensive study of fats/lipids and the pathways that make and break them, has gained traction because lipids are central both to Mycobacterium tuberculosis (Mtb) biology and to the human immune response (Cambier, Falkow and Ramakrishnan, 2014; Russell, Huang and VanderVen, 2019).

Lipidomics has been applied across diverse contexts: from analyzing the lipid-rich bacterial cell envelope and granuloma biology in experimental models, to profiling lipids in human samples such as blood/plasma, urine, sputum, and bronchoalveolar lavage (BAL). These investigations use established analytical technologies such as mass spectrometry (detects and measures molecules with high precision), chromatography (separates complex mixtures so individual lipids can be measured), Matrix-Assisted Laser Desorption/Ionization (MALDI), and nuclear magnetic resonance (NMR) (characterizes molecular structures) (Shivakoti *et al.*, 2022; Lyu *et al.*, 2024; Zhang *et al.*, 2025).

Despite rapid growth, the evidence base is fragmented: some studies are mechanistic (in vitro and ex vivo studies), others compare patients and controls (diagnostic), and others prognostic, track changes over treatment (monitoring/relapse). This heterogeneity increases the difficulty in judging the overall utility of lipidomics for TB biomarker discovery and has given rise to several ongoing debates in the field. For example, while some studies report strong diagnostic or prognostic performance for lipid-based biomarkers (Shivakoti *et al.*, 2022; Lyu *et al.*, 2024), others highlight limited reproducibility across populations and study designs (Zhang *et al.*, 2025). There is also discussion about whether lipid alterations observed in TB primarily reflect host immune-metabolic responses or pathogen-derived molecules, raising questions about specificity (Cambier, Falkow and Ramakrishnan, 2014; Russell, Huang and VanderVen, 2019). In addition, uncertainty persists over which biological specimen types offer the most reliable and scalable insights, and whether high-resolution platforms, particularly LC-MS and NMR, can be implemented sustainably in low-resource, high-burden settings, given recurring challenges with cost, maintenance, supply chains, power stability, and specialist staffing(Bell *et al.*, 2018; Lee, Chintalapudi and Badu-Tawiah, 2021; Letertre, Giraudeau and de Tullio, 2021; Cui *et al.*, 2025). These controversies further justify the need for a scoping review to systematically map how lipidomics has been applied in TB research and clarify where consensus and uncertainty currently lie.

Conceptually, this review is informed by three interlinked factors. First, lipids are not peripheral molecules in TB but central actors in both pathogen biology and host defense, positioning lipidomics as a uniquely relevant approach within the broader omics toolkit. Second, biomarker discovery in TB increasingly requires host-directed as well as pathogen-directed insights, and lipidomics offers a way of capturing these host-pathogen interactions in measurable form. Third, the existing evidence is highly heterogeneous, spanning different specimens, study models, and

analytic platforms, which creates uncertainty about where the most promising opportunities for biomarker development lie. These conceptual considerations underpin the decision to adopt a scoping review, designed to map fragmented and diverse literatures rather than test a single narrow hypothesis. At the same time, emerging approaches such as lipidomics must be evaluated not only for scientific merit but also for their potential to close gaps in diagnosis, prognosis, and treatment monitoring in high-burden settings.

Justification

A scoping review is warranted to systematically map how lipidomics has been applied across different sample types, study models, and research aims, in order to identify where evidence is clustered and where important gaps remain. Unlike systematic reviews, which suit narrow questions of intervention effectiveness, a scoping review captures and organizes the breadth of available evidence (Arksey and O'Malley, 2005; Levac, Colquhoun and O'Brien, 2010; Peters *et al.*, 2015). This approach is well-matched to an emerging, interdisciplinary area such as lipidomics in TB, where methods, populations, specimens, and applications vary widely.

By adopting a scoping review methodology, this project will:

- Provide a structured overview of lipidomics use in TB biomarker discovery to date;
- Identify methodological approaches and key lipid classes studied across human, animal, and in vitro work;
- Highlight evidence clusters and gaps to guide future research, including priorities for validation and clinical translation.

The review question also meets the FINER criteria (Hulley *et al.*, 2007), being feasible within the available resources, novel in addressing an underexplored omics domain, and highly relevant to TB research and policy in high-burden settings. As a secondary review it raises minimal ethical concerns, while its translational potential ensures interest to both scientific and public health communities.

Aim

To systematically map the evidence on how lipidomics has been applied in tuberculosis (TB) biomarker discovery, across different study types, specimen sources, and analytical approaches.

Objectives

1. To identify the range of specimen types and analytical platforms used in lipidomic investigations of TB.
2. To summarize the lipid classes and pathways reported in relation to TB across human, animal, and in vitro studies.
3. To map the applications of lipidomics in TB research, including diagnostic, prognostic, treatment-monitoring, and mechanistic studies.
4. To highlight knowledge clusters and research gaps that can guide future TB biomarker discovery efforts.

The PCC Framework based Review Question

- The population of the review question is human participants with TB, animal models of TB, and in vitro experimental systems in any global setting.

- The concept is two part, Lipidomics approaches (targeted or untargeted lipid profiling using mass spectrometry, chromatography, or NMR), and its application to biomarker discovery (diagnosis, prognosis, treatment response, or mechanistic insight).
- The context is tuberculosis infection and disease, in any global setting.

Review Question Narrative

Guided by established scoping review methodology (Arksey and O'Malley, 2005; Peters *et al.*, 2015), this review asks, how has lipidomics been applied in tuberculosis biomarker discovery across human, animal, and in vitro studies, and what specimen types, lipid classes, and applications have been reported to date?

Protocol Registration

To ensure methodological transparency and reproducibility, the protocol for this scoping review was prospectively archived (DOI **10.17605/OSF.IO/5EGMR**).

Eligibility Criteria

The following eligibility criteria have been selected using the PCC framework for scoping reviews.

Inclusion Criteria

Population

1. Human studies of any age group and clinical presentation of TB (pulmonary or extrapulmonary), animal models experimentally infected with Mycobacterium tuberculosis, and in vitro models (including macrophage or epithelial cell cultures) in any global setting.

Concept

2. Plasma, serum, urine, sputum, BAL, tissue biopsies, extracellular vesicles, or equivalent.
3. Studies applying lipidomic methods (untargeted or targeted profiling using LC-MS, GC-MS, NMR, MALDI, or equivalent technologies) to
4. TB biomarker discovery.

Context

5. Tuberculosis infection and disease, in any global setting.

Supplementary Inclusion Criteria

6. Observational (case-control, cohort, cross-sectional), interventional, and experimental studies.
7. Peer-reviewed publications and grey literature (including conference abstracts, theses, organizational reports) to reduce publication bias.
8. Publications in English, with no restrictions on year to capture the evolution of lipidomics in TB. Consideration will be given to the trade-off between likely bias versus feasibility of the research given time and other resource availability.

Exclusion Criteria

1. Studies in which lipidomics is not applied to TB.
2. Studies limited to other omics (including genomics, proteomics) without a lipidomic component.

3. Narrative reviews, commentaries, or editorials without primary data (though these may be screened for references).
4. Non-English publications (excluded due to feasibility and resource availability).

Search Strategy

Preliminary searches were conducted in PubMed using the string below (**Search Term** section). These yielded a manageable but diverse body of literature spanning human, animal, and in vitro studies. Identified work encompassed multiple specimen types (plasma, sputum, urine, tissue, and BAL) and employed a range of analytical platforms, including liquid chromatography-mass spectrometry (LC-MS), gas chromatography-mass spectrometry (GC-MS), and nuclear magnetic resonance (NMR). Exploratory use of broader terms such as *"omics AND tuberculosis"* retrieved an unfocused and excessively large set of records, while restricting the search to *"plasma lipidomics"* risked excluding valuable methodological and translational insights from other specimen types.

On this basis, the review scope was refined to include all lipidomics applications in tuberculosis biomarker discovery, while excluding studies where lipidomics was not a central component. Most eligible records were available in English, supporting the application of an English-language restriction. To enhance search sensitivity and optimize terminology, PubReMiner was employed in the preliminary scoping phase to identify recurring journals, authors, keywords, and publication trends. These outputs informed refinement of the PubMed search string and ensured sensitivity to emerging terminology in the lipidomics-TB field.

Building on this scoping phase, a comprehensive search strategy will be developed to identify all relevant studies on lipidomics in TB. Both peer-reviewed and grey literature will be included to

reduce publication bias. The strategy will combine controlled vocabulary (including Medical Subject Headings [MeSH] in PubMed/MEDLINE) with free-text keywords to maximize coverage and reproducibility.

Databases to be searched

This will include PubMed/MEDLINE, Embase, Web of Science, Scopus, CINAHL, ProQuest Dissertations & Theses, and Grey Literature (including WHO reports, TB Alliance, Gates Foundation reports).

Search Terms

The search strategy to be employed will combine both MeSH terms and free-text keywords using Boolean operators. An example search string has been included below:

("Tuberculosis"[Mesh] OR "Mycobacterium tuberculosis"[Mesh] OR tuberculosis[tiab] OR TB[tiab] OR "M. tuberculosis"[tiab]) AND ("Lipidomics"[Mesh] OR lipidomics[tiab] OR lipidomic[tiab] OR "lipid profiling"[tiab] OR "lipid analysis"[tiab] OR "lipid profile"[tiab] OR "lipid species"[tiab] OR "lipid mediator"[tiab] OR "mass spectrometry"[tiab]) AND ("Biomarkers"[Mesh] OR biomarker[tiab] OR diagnos*[tiab] OR prognos*[tiab] OR monitor*[tiab])*

Included above are the synonyms for tuberculosis and lipidomics have been included as well as a comprehensive use of the truncation operator, [*]. The search syntax above will be adapted for each database to ensure comprehensive retrieval of both indexed and newly published studies.

Screening process

Search results will be exported to reference management software (in particular Zotero), and duplicates removed. Screening will proceed in two stages: Firstly, title/abstract screening, and secondly, full text screening. Predefined eligibility criteria will guide screening decisions. To strengthen rigor, two reviewers (researcher and supervisor) will independently check the subset of articles and resolve discrepancies by discussion. The search strategy will be refined iteratively during preliminary runs to ensure it is both comprehensive and feasible.

Charting the Data

Data from eligible studies will be extracted systematically using a predefined comprehensive framework to ensure consistency and comparability. The charting process will capture both study characteristics and lipidomics-related findings relevant to TB biomarker discovery and translation. The draft framework is outlined below:

Table 1 Draft data extraction framework for the scoping review, outlining study characteristics, lipidomics methods, and reported findings in tuberculosis biomarker discovery.

**Variable**	**Details to Extract**
Bibliographic info	Author, year, country, journal
Study type	Human / animal / in vitro/ ex vivo
Population/sample	TB patients, controls, animal model, cell line
Specimen type	Plasma, serum, urine, sputum, BAL, tissue, EVs
Analytical platform	LC-MS, GC-MS, NMR, MALDI
Lipid classes studied	PC, PE, PI, TG, SM, Cer, LPC etc.
Application	Diagnostic, prognostic, treatment monitoring, mechanistic/pathogenesis
Key findings	Lipids significantly altered, biomarker performance (AUC, fold changes, pathways)
Reported gaps	Validation needs, scalability issues, under-studied populations and geographical locations, translation potential

BAL = bronchoalveolar lavage; EVs = extracellular vesicles; LC–MS = liquid chromatography–mass spectrometry; GC–MS = gas chromatography–mass spectrometry; NMR = nuclear magnetic resonance; MALDI = matrix-assisted laser desorption/ionization; AUC = area under the curve; Example lipid classes:

PC – Phosphatidylcholine; PE – Phosphatidylethanolamine; PI – Phosphatidylinositol; TG – Triacylglycerol; SM – Sphingomyelin; Cer – Ceramide; LPC – Lysophosphatidylcholine.

This framework will remain flexible and may be refined iteratively during the review to accommodate unanticipated variables. Extracted data will be recorded in a standardized spreadsheet, enabling both descriptive numerical summaries, and thematic synthesis in subsequent analysis.

Data Analysis & Synthesis

Descriptive numerical summaries will be provided, including the number of studies, study types, specimen sources, analytical platforms, and lipid classes investigated. A thematic synthesis will then be undertaken, with findings grouped according to:

- Specimen type – plasma, sputum, urine, tissue, BAL, EVs.
- Study type – human, animal, in vitro, ex vivo.
- Lipid class or pathway – sphingolipids, glycerophospholipids, sterols, glycerolipids, diacylglycerols.
- Biomarker application – diagnostic, prognostic, treatment monitoring, mechanistic.

This dual approach will provide both a numerical overview and a thematic mapping of the evidence. Results will be presented in tabular and visual formats, including evidence mapping tables and graphical displays such as bubble plots (which display study volume relative to two variables) and Sankey diagrams (flow diagrams showing connections between specimen types, lipid classes, and applications).

Where reported, quantitative performance metrics (including AUCs for diagnostic accuracy, fold-change differences in lipid levels) will be extracted and summarized. The analysis and synthesis plan will follow the framework for scoping reviews first proposed by Arksey and O'Malley (2005), refined by Levac et al. (2010), and Peters et al. (2015), and reported in accordance with the PRISMA-ScR checklist (Tricco *et al.*, 2018). A PRISMA-ScR flow diagram will be used to depict study selection, ensuring methodological consistency, transparency, and reproducibility.

Timeframe

The scoping review is planned over a 12-week schedule, incorporating sequential and overlapping phases designed to balance methodological rigor with feasibility and ensure timely completion within the project's allocated period.

Table 2 Proposed 12-week timeline for the scoping review, outlining sequential activities and expected outputs.

	**Activity**	**Weeks**												**Milestones/Outputs**
		**1**	**2**	**3**	**4**	**5**	**6**	**7**	**8**	**9**	**10**	**11**	**12**	
1.	Preliminary scoping searches; protocol development													Refined PCC question and protocol draft
2.	Database and grey literature searches; initial screening of titles/abstracts													Search results database; shortlist of potential studies
3.	Full-text screening; application of inclusion/exclusion criteria													Final list of included studies
4.	Data extraction and charting													Populated extraction framework (spreadsheet)
5.	Data synthesis and drafting of results													Draft tables, evidence maps, narrative synthesis

6.	Final review, editing, and submission													Completed project assignment

PCC = Population, Concept, Context framework used to structure the review question.

References

1. Tricco, A.C. *et al.* (2018) "PRISMA Extension for Scoping Reviews (PRISMA-ScR): Checklist and Explanation," *Annals of Internal Medicine*, 169(7), pp. 467–473. Available at: https://doi.org/10.7326/M18-0850.
2. Bell, L. *et al.* (2018) "Challenges and Opportunities for Biological Mass Spectrometry Core Facilities in the Developing World," *Journal of Biomolecular Techniques: JBT*, 29(1), pp. 4–15. Available at: https://doi.org/10.7171/jbt.18-2901-003.
3. Lee, S., Chintalapudi, K. and Badu-Tawiah, A.K. (2021) "Clinical Chemistry for Developing Countries: Mass Spectrometry," *Annual review of analytical chemistry (Palo Alto, Calif.)*, 14(1), pp. 437–465. Available at: https://doi.org/10.1146/annurev-anchem-091520-085936.
4. Peters, M.D.J. *et al.* (2015) "Guidance for conducting systematic scoping reviews," *JBI Evidence Implementation*, 13(3), p. 141. Available at: https://doi.org/10.1097/XEB.0000000000000050.
5. Cambier, C.J., Falkow, S. and Ramakrishnan, L. (2014) "Host Evasion and Exploitation Schemes of Mycobacterium tuberculosis," *Cell*, 159(7), pp. 1497–1509. Available at: https://doi.org/10.1016/j.cell.2014.11.024.
6. Russell, D.G., Huang, L. and VanderVen, B.C. (2019) "Immunometabolism at the interface between macrophages and pathogens," *Nature Reviews Immunology*, 19(5), pp. 291–304. Available at: https://doi.org/10.1038/s41577-019-0124-9.
7. Lyu, L. *et al.* (2024) "Individualized lipid profile in urine-derived extracellular vesicles from clinical patients with Mycobacterium tuberculosis infections," *Frontiers in Microbiology*, 15, p. 1409552. Available at: https://doi.org/10.3389/fmicb.2024.1409552.
8. Letertre, M.P.M., Giraudeau, P. and de Tullio, P. (2021) "Nuclear Magnetic Resonance Spectroscopy in Clinical Metabolomics and Personalized Medicine: Current Challenges and Perspectives," *Frontiers in Molecular Biosciences*, 8, p. 698337. Available at: https://doi.org/10.3389/fmolb.2021.698337.
9. Ong'ang'o, J.R. *et al.* (2025) "Persistently high TB prevalence in Nairobi County neighbourhoods, 2015–2022," *PLOS Global Public Health*, 5(2), p. e0003849. Available at: https://doi.org/10.1371/journal.pgph.0003849.
10. Zhang, C. *et al.* (2025) "Plasma lipidomic analysis reveals distinct lipid alterations in patients with pulmonary tuberculosis," *European Journal of Medical Research*, 30, p. 566. Available at: https://doi.org/10.1186/s40001-025-02835-6.
11. Levac, D., Colquhoun, H. and O'Brien, K.K. (2010) "Scoping studies: advancing the methodology," *Implementation Science*, 5(1), p. 69. Available at: https://doi.org/10.1186/1748-5908-5-69.
12. Arksey, H. and O'Malley, L. (2005) "Scoping studies: towards a methodological framework," *International Journal of Social Research Methodology*, 8(1), pp. 19–32. Available at: https://doi.org/10.1080/1364557032000119616.
13. Cui, Y. *et al.* (2025) "The Evolving Landscape of Host Biomarkers for Diagnosis and Monitoring of Tuberculosis," *Biomedicines*, 13(9), p. 2076. Available at: https://doi.org/10.3390/biomedicines13092076.
14. Hulley, S.B. *et al.* (2007) *Designing Clinical Research*. 3rd ed. Philadelphia, PA: Lippincott Williams & Wilkins.
15. WHO (2024) *Global Tuberculosis Report 2024*. Geneva: World Health Organization. Available at: chrome-extension://efaidnbmnnnibpcajpcglclefindmkaj/https://iris.who.int/bitstream/handle/10665/379339/9789240101531-eng.pdf?sequence=1 (Accessed: April 21, 2025).

55. Shivakoti, R. et al. (2022) "Host lipidome and tuberculosis treatment failure," The European RespiratoryJournal, 59(1), p. 2004532. Available at: https://doi.org/10.1183/13993003.04532-2020.
