# Supplementary material for: Closing the Translational Gap in Tuberculosis Lipidomics for Resource-Constrained Settings
Source: Metabolites. 2026 Sep 24;16(10):708. doi: 10.3390/metabo16100708 (PMC13706928; doi:10.3390/metabo16100708)
Supplement: Supplementary file 1 [file metabolites-16-00708-s001.zip › metabolites-4488496-Table S1 Full list of included studies (n = 48).pdf]

*Supplementary Table S1. Full list of included studies (n = 48)*

| No. | Paper_ID | Geog_Origin | Study_Focus | Pop_Age | Study_Design | Platform | Specimen | Lipid_Classes and Candidate Biomarkers Identified | Identified_Gap |
|---|---|---|---|---|---|---|---|---|---|
| 1 | Zhu et al., 2026 | LMIC (China) [53] | Diagnosis | Adult (18+) | Multi-omics observational | LC-MS/MS | Plasma/Serum | Glycerophospholipids (PC, PE), Sphingolipids (SM, Cer), Fatty Acids | Lack of integrated multi-omics biomarkers that are validated across independent cohorts for early diagnosis. |
| 2 | Brandenburg et al., 2022 [61] | HIC (Germany) | Diagnosis / Treatment Monitoring | Adult (18+) | Multi-omics observational | LC-MS/MS (Orbitrap Q Exactive Plus) | Mixed (PBMCs, Sputum, Lung Tissue, Isolates) | Phospholipids (PI, PE, PG), TSA-containing species | Culture-based burden assessment is too slow (2 months); need for rapid, culture-free markers of bacterial load. |
| 3 | Zhao et al., 2025 [65] | LMIC (China) | Diagnosis (TB-CAD Comorbidity) | Adult (18+) | Case-control / diagnostic | LC-MS/MS (UPLC-MS/MS) | Plasma/Serum | Sphingolipids, Glycerolipids, Glycerophospholipids (CE, PC) | Standard lipid parameters (TG, TC, LDL-c, HDL-c) are insufficient for diagnosing TB-CAD comorbidity. |
| 4 | Collins et al., 2021 [45] | Mixed (HIC: USA; LMIC: Georgia, South Africa) | Pathogenesis / Treatment Response | Adult (18+) | Multi-omics observational | LC-MS/MS (High-resolution metabolomics /lipidomics) | Plasma/Serum | Lyso-phospholipids, Arachidonic acid, Proinflammatory Eicosanoids | Delayed effective treatment for MDR-TB allows proinflammatory metabolic cascades (succinate/itaconate shift) to perpetuate. |

| | | | | | | | | | |
|---|---|---|---|---|---|---|---|---|---|
| 5 | Sun et al., 2025 [46] | LMIC (China) | Pathogenesis / Diagnosis | Pediatric (<14 years) | Cross-sectional / Multi-omics | LC-MS/MS (UHPLC-MS/MS) | Mixed (Plasma and Stool) | LysoPC, Sphingolipids, Glycerolipids, Glycerophospholipids | Interactive mechanisms between gut microbiome and plasma lipids in children are not well explored compared to adults. |
| 6 | Wood et al., 2017 [41] | LMIC (African cohort via WHO/FIND) | Pathogenesis / Diagnosis | Adult (Average 47.5 years) | Case-control / diagnostic | LC-MS/MS (Shotgun ESI-Orbitrap) | Plasma/Serum | Phosphatidylcholines (PC), Lysophosphatidylcholines (LPC), Phosphatidylglycerols (PG) | Etiology of decreased LPC levels in TB had not been explored, and critical lung surfactant lipids (PG) were previously unexamined. |
| 7 | Zhang et al., 2025 [10] | LMIC (China - Jinjiang City) (1, 2) | Diagnosis / Biomarker Discovery (1) | Adult (Average 44.88 years) (4) | Case-control (1) | LC-MS/MS (Waters UPLC coupled with QTRAP 4500) (3) | Plasma/Serum (1) | PE, PC (elevated); TAG, CER, HCER, PG (reduced) (1, 5) | Inter-study heterogeneity due to variations in analytical platforms, cohort characteristics, and biological matrices (2, 10). |
| 8 | Luo et al., 2020 [31] | LMIC (China - Wuhan) | Diagnosis (TPE vs. Malignancy) | Adult (Average 47-60 years) | Multi-omics observational | LC-MS/MS (Shimadzu UHPLC with Q-Trap 5500) | Other (Pleural Effusion Extracellular Vesicles) | Phosphoglycerolipids (PC, PE, PS), Sphingolipids (SM, Cer) | Metabolic characteristics of EV subpopulations in pleural effusion and their diagnostic roles were previously unknown. |

| | | | | | | | | | |
|---|---|---|---|---|---|---|---|---|---|
| 9 | Wang et al., 2022 [52] | LMIC (China - Chengdu) | Safety / Prediction (ATB-DILI) | Adult (Average 33-39 years) | Cohort (Prospective) | LC-MS/MS (Waters 2D UPLC coupled with Q Exactive HF) | Urine | Bile acids (Glycocholate, Taurolithocholic acid 3-sulfate) | Lack of predose (premedication) data and predictive models for identifying individuals at risk for ATB-DILI. |
| 10 | Andreas et al., 2020 [39] | Mixed (LMIC: Gambia; HIC: UK) | Diagnosis | Pediatric (<15 years) | Case-control | Mixed (NMR and LC-MS/MS) | Plasma/Serum | Ceramides; Gangliosides (GM3); Triacylglycerides; Hexosylceramides | Sparse pediatric biomarker data (only 6% of TB literature); models need validation in HIV co-infected populations. |
| 11 | Chen et al., 2020 [22] | LMIC (South Africa - Cape Town) | Diagnosis / Screening | Adult (18+) | Case-control | LC-MS/MS (Direct Infusion Orbitrap MS) | Other (Exhaled Breath Aerosol/Particles) | Phospholipids (DPPC); Glycolipids | Standard sputum tests lack sensitivity; need for rapid, non-invasive screening feasible in high-burden settings. |
| 12 | Mosquera-Restrepo et al., 2022 [16] | LMIC (Colombia - Medellín) | Diagnosis / Treatment Monitoring | Mixed (Adult and Pediatric) | Case-control / Cohort (Longitudinal) | Mixed (NMR and SFC-HRMS) | Other (Exhaled Breath Condensate) | LAM; PIMs; Sulfoglycolipids; PDIM; Mycolic Acids | Sputum-based tests are inefficient for children and paucibacillary patients; need for non-sputum POC diagnostics. |

| | | | | | | | | | |
|---|---|---|---|---|---|---|---|---|---|
| 13 | Fong et al., 2023 [50] | LMIC (China - Guangzhou) | Diagnosis (TBM) | Adult (Ages 11+, mean 41.3) | Case-control | GC-MS | Cerebrospinal fluid (CSF) | Tuberculostearic acid (TBSA) | Low sensitivity of traditional TBM tests; TBSA results are often inconsistent when used alone without integrated scoring. |
| 14 | Wang et al., 2024 [37] | LMIC (China - Shanghai) | Diagnosis / Differential Diagnosis (MTB vs. NTM) | Adult (18-75 years) | Multi-omics observational | LC-MS/MS (TripleTOF 6600) | Plasma/Serum | FAHFAs; PCs; LPCs; PEs; LPEs; DGDGs; Ceramides | Specific impact of host immune status on NTM/MTB progression is unclear; need for reliable markers to differentiate MTB from SGM/RGM. |
| 15 | Chen et al., 2021 [58] | LMIC (China - Zhejiang/Guangdong) | Diagnosis / Treatment Monitoring | Adult (Mean age ~32-34 years) | Cohort / Longitudinal (Followed for 6 months) | LC-MS/MS (UPLC-QTRAP 6500+) | Plasma/Serum | Phospholipids (LPA, PC, LPE); Sphingolipids (SM, Cer); Glycerides (TG, DG, MG) | Lack of laboratory standards and accurate biomarkers for evaluating TB treatment efficacy. |
| 16 | Shivakoti et al., 2022 [55] | LMIC (India - Pune and Chennai) | Treatment Outcome (Failure prediction) | Adult (>= 18 years) | Case-control (Nested within a prospective cohort) | LC-MS/MS (LC-QTOF and UPLC-MS/MS) | Plasma/Serum (Heparin Plasma) | Cholesteryl esters; Ceramides; Triglycerides; Oxylipins; Phospholipids | Prospective association of baseline host lipids with future treatment failure not well characterized. |

| | | | | | | | | | |
|---|---|---|---|---|---|---|---|---|---|
| 17 | Reinecke et al., 2026 [20] | LMIC (South Africa - Western Cape) | Pathogenesis / Characterization | Adult (Mean age 47.35) | Case-control | GCxGC-TOFMS | Urine | Dicarboxylic acids; Fatty acid catabolites | TB-T2DM comorbidity is poorly understood metabolically despite its rising global prevalence. |
| 18 | Wang et al., 2022 (Guangdong) [51] | LMIC (China - Guangdong Province) | Diagnosis / Prognosis (LTBI vs. Active TB) | Adult (18-59 years) | Cohort / Longitudinal (Followed 6 months) | Mixed (GC-MS and LC-MS/MS) | Plasma/Serum | Fatty acids (3-oxopalmitic acid); Bile acids (Sulfolithocholic acid) | Lack of effective biomarkers to distinguish LTBI from active PTB and lack of uniform laboratory standards for patient discharge. |
| 19 | Feng et al., 2015 [17] | LMIC (China - Tianjin) | Diagnosis (Active TB vs. Non-TB lung diseases) | Adult (Mean age 48.3 years) | Case-control | LC-MS/MS (UPLC-MS/MS) | Plasma/Serum (Serum) | Lysophosphatidylcholines (lysoPC); Fatty acids | Lack of biomarkers capable of distinguishing active TB from general inflammatory responses or other specific lung diseases. |
| 20 | Kim et al., 2024 [44] | HIC (USA) | Pathogenesis (Brain Lipidome Dysregulation) | Pediatric (Rabbit Model) | Experimental / laboratory | Mixed (MALDI-MSI and LC-MS/MS) | Brain Tissue | Sphingolipids; Glycerophospholipids; Sterols; Oxysterols | The effect of M. tuberculosis infection on the young or adult brain lipidome has not been studied; lack of spatial mapping in brain lesions. |

| | | | | | | | | | |
|---|---|---|---|---|---|---|---|---|---|
| 21 | Anh et al., 2024 [32] | HIC (South Korea - Seoul) | Treatment Monitoring / Diagnosis | Adult (18+) | Cohort / Longitudinal (Followed through completion) | LC-MS/MS (Shimadzu Nexera LC + X500R QTOF) | Plasma/Serum (Plasma) | Glycerophospholipids; Lysolipids (LPC, LPE, LPI); Acyl-carnitines | Specific biomarkers for assessing the efficacy of anti-TB treatment are lacking. |
| 22 | Vrieling et al., 2018 [38] | LMIC (South Africa - Cape Town) | Pathogenesis / TB-DM Interaction | Adult (18-70 years) | Case-control | NMR (1H NMR spectroscopy) | Plasma/Serum | Sphingomyelins; VLDL-triglycerides; Cholesteryl esters; Phosphoglycerides | The molecular foundations of the increased risk and worsened outcomes in TB-DM comorbidity are unclear. |
| 23 | Liu et al., 2021 [25] | LMIC (China - Hangzhou) | Diagnosis (TBPE vs. Malignant Effusion) | Adult (Mean age 48.8) | Case-control / diagnostic | GC-MS | Other (Pleural Fluid) | Fatty acids (Stearic acid); Fatty acid amides (Oleamide) | Clinical differentiation of TBPE from MPE is challenging; existing tests like IGRA and Xpert have limited sensitivity in pleural fluid. |
| 24 | Collins et al., 2018 [26] | LMIC (Georgia - Tbilisi) | Diagnosis (Biomarker Discovery) | Adult (Median age 27) | Case-control | LC-MS (High-resolution Orbitrap) | Plasma/Serum | Phosphatidylglycerols (PG); Lysophosphatidylinositols (Lyso-PI); Acylphosphatidylinositol mannosides | Standard human databases lack Mtb-derived metabolites; need for robust blood-based biomarkers for point-of-care use. |
| 25 | Chen et al., 2022 [36] | LMIC (China - Beijing) | Diagnosis (Osteoarticular TB) | Adult (Median age 46) | Case-control / diagnostic | LC-MS/MS (Q Exactive Orbitrap) | Plasma/Serum | Phosphatidylcholines (PC); Sphingomyelins (SM); Ceramides (Cer); PE; Fatty acids | Low specificity of current detection methods for osteoarticular TB leading to high misdiagnosis rates. |

| | | | | | | | | | |
|---|---|---|---|---|---|---|---|---|---|
| 26 | Frediani et al., 2014 [42] | LMIC (Georgia - Tbilisi) | Diagnosis / Pathogenesis | Adult (18+) | Case-control | LC-MS (High-resolution Orbitrap) | Plasma/Serum | Resolvins (RvD1, RvD2); Trehalose-6-mycolate; Phosphatidylinositol | Lack of biomarkers to predict transition from latent to active TB or to monitor efficacy of anti-TB drugs. |
| 27 | Vinhaes et al., 2019 [19] | LMIC (China - Zhengzhou) | Treatment Response / Pathogenesis (PTB vs. EPTB) | Adult (Median age 27) | Cohort / Longitudinal (Followed for 24 weeks) | EIA (Targeted Enzyme Immunoassay for lipids) | Plasma/Serum | Eicosanoids (Prostaglandins: PGE2, PGF2α; Lipoxins: LXA4, 15-EPI-LXA4) | Lack of biomarkers for disease staging and treatment outcome prediction; persistent inflammatory imbalance post-treatment is poorly understood. |
| 28 | Pavan Kumar et al., 2019 [40] | LMIC (India - Chennai) | Pathogenesis / TB-DM Interaction | Adult (Median age 39–47) | Case-control (with 6-month longitudinal follow-up) | ELISA (Targeted) | Plasma/Serum | Eicosanoids (Lipoxins: LXA4, 15-epi-LXA4; Leukotrienes: LTB4; Prostaglandins: PGE2) | Lack of detailed investigation into how eicosanoids relate to lung pathology and bacterial burden in TB-DM. |
| 29 | Kagemann et al., 2025 [33] | LMIC (India - Puducherry/Chennai) | Pathogenesis / Treatment Response | Adult (>=18 years) | Cohort / Longitudinal (Baseline, 1 month, 6 months) | LC-MS/MS (Tandem mass spectrometry) | Plasma/Serum | Glycerophospholipids (LysoPI, PE, PI, LPC, LPE, LPA, LPS); Sphingolipids (Cer, Sphingosine) | Effect of disease severity on metabolite dynamics is understudied; previous severity classifications relied solely on CXR and lacked sputum smear indicators. |

| | | | | | | | | | |
|---|---|---|---|---|---|---|---|---|---|
| 30 | Arriaga et al., 2022 [23] | LMIC (Brazil and South Africa) | Treatment Response / TB-DM Interaction | Adult (18+) | Cohort / Longitudinal (Multi-site prospective observational) | LC-MS/MS (UPLC/ESI-MS/MS) | Urine | Eicosanoids (Prostaglandins: PGE-M, TN-E, PGD-M, PGI-M; Thromboxanes: 11dTxB2; Leukotrienes: LTE4) | Unknown associations between diabetes-related lipid mediator changes and M. tuberculosis clearance during anti-TB treatment. |
| 31 | Wang et al., 2017 [34] | LMIC (China - Kunming) | Diagnosis (TPE vs. Malignant vs. Transudative) | Adult (Mean age 53.2) | Case-control / diagnostic | NMR (1H NMR) | Other (Pleural Fluid) | Low-density lipoprotein (LDL); Phospholipids | Lack of metabolic profiling for pleural effusion; morphological similarities between etiologies lead to diagnostic difficulty. |
| 32 | Lyu et al., 2024 [7] | LMIC (China - Beijing) | Diagnosis (TB vs. LTBI) | Adult (18+) | Case-control / diagnostic | LC-MS (UPLC-TQMS) | Other (Urinary EVs) | FFA; PC; SM; PE; DAG; MAG; CE; Cer | Lipidome of urine-derived EVs from TB patients previously unreported; difficulty in identifying biomarkers in raw urine due to component complexity. |

| | | | | | | | | | |
|---|---|---|---|---|---|---|---|---|---|
| 33 | Dai et al., 2017 [27] | LMIC (China - Hangzhou) | Diagnosis (TBM vs. Other Meningitis) | Adult (Mean age 47.6) | Case-control / diagnostic | LC-MS (UHPLC-QTOF-MS) | CSF (Cerebrospinal Fluid) | PE; PA; PC; PG; Fatty acids; Bile acids; Stearoylcarnitine | Scarcity of CSF metabolome data for differentiating TBM from other meningitis types; existing tests lead to delayed intervention. |
| 34 | Chen et al., 2022 (Sci Rep) [70] | LMIC (South Africa - Cape Town) | Diagnosis | Adult (Mean age 29–33) | Case-control | LC-MS/MS (LTQ Orbitrap) | Other (Exhaled Breath Aerosol) | Phosphatidylinositol (PI); Phosphatidylserine (PS); Ceramides (Cer); DG; PC; TG | Need for non-sputum specimen collection methods; metabolic origins of VOCs are poorly understood compared to NOCs. |
| 35 | Llibre et al., 2022 [48] | LMIC (South Africa - Worcester) | Pathogenesis / Host-Directed Therapy | Adult (Median age 33) | Case-control (with longitudinal follow-up) | LC-MS/MS (UPLC-MS/MS) | Plasma/Serum | Pregnane steroids; Sphingomyelins | Lack of comprehensive understanding of how Mtb impacts host immune-metabolic pathways; triggers of IL-1ra in TB remain unknown. |
| 36 | Pal et al., 2017 [21] | LMIC (India - New Delhi) | Basic Pathogenesis / Drug Resistance | N/A (Laboratory clinical isolates) | Experimental / laboratory | LC-MS/MS (Triple quadrupole tandem MS) | Cell Lysate (MTB bacterial cells) | Mycolic acids; PIMs; Phosphatidylinositol; Cardiolipin; Triacylglycerides | Lack of a comparable lipidomic resource between drug-sensitive and resistant strains to understand MDR acclimatization. |

| | | | | | | | | | |
|---|---|---|---|---|---|---|---|---|---|
| 37 | Anh et al., 2023 [59] | HIC (South Korea - Busan/Seoul) | Treatment Monitoring / Evaluation | Adult (18+) | Cohort / Longitudinal (Prospective 6-month follow-up) | LC-MS (UPLC-QToF MS) | Plasma/Serum (Plasma) | PC; MG; TG; CE; SM; Ether-linked PEs | Microbiological and radiological monitoring is inconsistent after treatment starts; need for non-sputum molecular alternatives. |
| 38 | Todorova et al., 2023 [24] | LMIC (Bulgaria) | Diagnosis / Prognosis (Differentiating stages) | Adult (18+) | Case-control | ELISA | Plasma/Serum | Eicosanoids (PGE2, LXA4) | Need for biomarkers to predict the course of MTB infection and the necessity of specific therapy following recent contact. |
| 39 | Beukes et al., 2023 [49] | LMIC (South Africa) | Diagnosis / Confounder Analysis | Adult (20–60 years) | Cross-sectional | GCxGC-TOFMS | Sputum | Lysophosphatidic acids (LPA); Fatty acids; Cholesterol | High individual variation in TB metabolome studies; lack of biomarkers that are validated independent of sex and HIV status. |
| 40 | Huang et al., 2020 [47] | LMIC (China - Jiaxing/Shaoxing) | Diagnosis (MDR-TB vs. DS-TB) | Adult (Mean age 37–41) | Case-control / diagnostic | LC-MS (UPLC-Q-TOF-MS) | Plasma/Serum (Serum) | Phosphatidylcholines (MG3P, DPPC); Fatty acids (Caprylic acid) | Lack of rapid diagnostics for MDR-TB; current DST takes 3–8 weeks, obstructing timely drug regimen adjustment. |

| | | | | | | | | | |
|---|---|---|---|---|---|---|---|---|---|
| 41 | Han et al., 2021 [57] | LMIC (China - Hangzhou/Yangjiang/Shaoxing) | Diagnosis (TB vs. LC vs. CAP) | Adult (Mean age 41.6) | Case-control / diagnostic | LC-MS/MS (UPLC-MS/MS) | Plasma/Serum | Phosphatidylcholines (PC); Cholesteryl esters (CE); Sphingomyelins (SM) | Difficult to diagnose TB in time based on bacteriological methods alone; host lipid metabolism in plasma not fully elucidated. |
| 42 | Li et al., 2024 [30] | LMIC (China - Chengdu) | Safety / Prediction (ATB-DILI) | Adult (Mean age 37–39) | Cohort / Longitudinal (Prospective with multiple timepoints) | LC-MS/MS (Discovery HD4™ and UPLC-MS/MS) | Plasma/Serum (Plasma) | Fatty acids; Bile acids; Carnitines; Ketone bodies | Cross-sectional data has limited predictive ability; lack of insights into how metabolite levels change dynamically with DILI progression. |
| 43 | Shivakoti et al., 2020 [29] | LMIC (India - Pune) | Pathogenesis / TB-DM Interaction | Adult (Median age 43) | Case-control (Nested cross-sectional) | LC-MS/MS (Targeted lipid mediator profiling) | Plasma/Serum (Serum) | SPMs (Lipoxins, Maresins, Resolvins); Pro-inflammatory (LTB4, PGE2, LTE4) | Lack of characterization of circulating lipid mediators beyond a few common species in the context of TB-DM comorbidity. |
| 44 | Zhu et al., 2026 [53] | LMIC (China - Henan/Zhejiang) | Diagnosis / Treatment Monitoring (Cure Assessment) | Adult (Median age 32–34) | Multi-omics observational (Integrated with Mendelian Randomization) | LC-MS/MS (UPLC-MS/MS and DIA-Proteomics) | Plasma/Serum | Phosphatidylcholines (PC); Triglycerides (TG); PEs; SMs | Lack of objective and quantifiable laboratory evaluation systems for treatment efficacy and confirming cure status. |

| | | | | | | | | | |
|---|---|---|---|---|---|---|---|---|---|
| 45 | Lau et al., 2015 [35] | HIC (Hong Kong) | Diagnosis (Differential Diagnosis vs. CAP) | Adult (18+) | Case-control / diagnostic | LC-MS (UHPLC-ESI-QTOFMS) | Plasma/Serum | Ceramides; Cholesterol metabolites (Cholesterol sulfate); Eicosanoids (12(R)-HETE) | Previous studies lacked non-TB infectious disease controls; potential of metabolic profiles to distinguish TB from other bacterial infections was unknown. |
| 46 | Daniel et al., 2025 [43] | LMIC (India - Chennai/Pune) | Prognosis (Risk of Progression) | Mixed (Adults and Children) | Cohort / Longitudinal (2-year prospective follow-up) | LC-MS/MS (QTRAP 6500) | Other (QuantiFERON Supernatants) | Phosphatidylcholines (PC O-); Diglycerides (DG); Fatty acids | Existing tests (IGRA/TST) cannot predict the dynamic TB disease spectrum or identify who will actually progress to active disease. |
| 47 | Wang et al., 2023 [28] | LMIC (China - Hangzhou) | Pathogenesis / TB-DM Interaction | Adult (18–65 years) | Multi-omics observational (Transcriptomics + Metabolomics) | LC-MS (Untargeted) | Mixed (Whole Blood/Serum) | Glycerophospholipids (LysoPC, LysoPE); Triacylglycerols (TG); PC | Molecular mechanisms underlying the susceptibility of DM patients to PTB remain unknown; lack of tools for early prediction. |
| 48 | Liu et al., 2024 [18] | LMIC (China - Hangzhou/Shanghai) | Diagnosis / Phenotyping (Drug Resistance) | Adult (18+) | Case-control / diagnostic | NPELDI-MS (Nanoparticle-enhanced MS) | Plasma/Serum | PC; LysoPC; Cholesteryl esters (CE); TG; Monoglycerides (MG); Ceramides (Cer) | Lack of integrated, rapid, and cost-effective methods for simultaneous TB diagnosis and drug-resistance phenotyping. |
