# Supplementary material for: Closing the Translational Gap in Tuberculosis Lipidomics for Resource-Constrained Settings
Source: Metabolites. 2026 Sep 24;16(10):708. doi: 10.3390/metabo16100708 (PMC13706928; doi:10.3390/metabo16100708)
Supplement: Supplementary file 1 [file metabolites-16-00708-s001.zip › Table S2 Complete database search log.pdf]

Supplementary Table S2. Complete database search log

Database	Date Searched	Population/Concept Block	Full Search String	Records Retrieved	Export Format	Notes
PubMed	19/03/2026	Tuberculosis AND lipidomics/lipid profiling, including mixed metabolomics with lipid terms	("Tuberculosis"[Mesh] OR "Mycobacterium tuberculosis"[Mesh] OR tuberculosis[tiab] OR tuberculous[tiab] OR "Mycobacterium tuberculosis"[tiab] OR "M. tuberculosis"[tiab]) AND ("Lipidomics"[Mesh] OR lipidomic*[tiab] OR lipidom*[tiab] OR "lipid profiling"[tiab] OR "lipid profile*"[tiab] OR "targeted lipidomics"[tiab] OR "untargeted lipidomics"[tiab] OR "shotgun lipidomics"[tiab] OR (metabolom*[tiab] AND (lipid*[tiab] OR phospholipid*[tiab] OR sphingolipid*[tiab] OR ceramide*[tiab] OR triglycerid*[tiab] OR cholesteryl ester*[tiab] OR lysophosphatidylcholine*[tiab] OR phosphatidylcholine*[tiab] OR oxylipin*[tiab]))) AND english[lang] NOT (review[Publication Type] OR systematic review[ti] OR review[ti] OR editorial[Publication Type] OR comment[Publication Type])	257	NBIB	Final locked PubMed core search after iterative refinement. Retrieved 257 records. Demonstrated strong sentinel recovery, with 18 sentinel studies identified within the first 40 records. Additional missing sentinel studies to be recovered through citation tracking and known-item searching.

PubMed	19/03/2026	Citation tracking from sentinel studies	N/A	75	NBIB	Citation tracking was conducted from 19/03/2026 to 26/03/2026 through backward and forward citation chaining of records identified in the primary PubMed search. This yielded 77 additional publications for deduplication and eligibility screening, and completes the citation-tracking stage for this review update.

PubMed	19/03/2026	Citation tracking from sentinel studies	N/A	12	RIS	Citation tracking was conducted from 19/03/2026 to 26/03/2026 through backward and forward citation chaining of records identified in the primary PubMed search. This yielded 12 additional publications for deduplication and eligibility screening, and completes the citation-tracking stage for this review update.

G_Scholar	26/03/2026	Tuberculosis AND lipidomics	"tuberculosis" "plasma lipidomics"	136	EndNote	The refined Google Scholar search string “tuberculosis” “plasma lipidomics” yielded 136 results, of which 3 were highly relevant on title and abstract screening, and was retained as a focused supplementary search. Relevant records were saved to Google Scholar My Library and exported by label for import into the review workflow; only highly relevant records were exported for deduplication and eligibility assessment.

G_Scholar	26/03/2026	Tuberculosis AND lipidomics AND Biomarkers	tuberculosis "plasma lipidomics" biomarkers	315	EndNote	The refined Google Scholar search string “tuberculosis” “plasma lipidomics” biomarkers yielded 126 results and showed improved topical relevance on preliminary screening. It was retained as a focused supplementary search and 7 relevant records were exported for deduplication and eligibility assessment.

Europe_PMC	26/03/2026	Tuberculosis AND lipidomics AND Biomarkers	tuberculosis AND "lipidomics" AND "Biomarker*" AND (((SRC:MED OR SRC:PMC OR SRC:AGR OR SRC:CBA) NOT (PUB_TYPE:"Review")))	277	RIS	The Europe PMC search was undertaken as a supplementary bibliographic search using a refined tuberculosis–lipidomics–biomarker strategy with no limits applied. Earlier broader searches were iteratively narrowed because they retrieved a high proportion of nonspecific records. The final search yielded 277 records, of which 20 were retained as most relevant on title and abstract screening for deduplication and eligibility assessment within the review workflow.

OpenALex	26/03/2026	Tuberculosis AND Lipidomics	tuberculosis lipidomics	126	BibTeX/RIS/NBIB	The OpenAlex search string tuberculosis lipidomics yielded 126 works and was retained as a broader supplementary search. Although the retrieval included both host-focused and broader mycobacterial lipidomics literature, 16 records were judged relevant on title and abstract screening and were retained for deduplication and eligibility assessment within the review workflow.

OpenALex	26/03/2026	Tuberculosis AND Lipidomics AND Plasma	tuberculosis "plasma lipidomics"	2	BibTeX/RIS/NBIB	The OpenAlex search string tuberculosis "plasma lipidomics" yielded 2 works, both of which were directly relevant on title and abstract screening. It was retained as a highly specific supplementary search for import into the review workflow, deduplication, and eligibility assessment.

AJOL	26/03/2026	N/A	N/A		N/A	AJOL was searched as a supplementary regional source using simplified tuberculosis–lipidomics keyword combinations. No studies were retrieved from the search lines tested, and AJOL did not contribute additional records to the review workflow.
